# Inhibition of p38 MAPK activity leads to cell type-specific effects on the molecular circadian clock and time-dependent reduction of glioma cell invasiveness

**DOI:** 10.1186/s12885-017-3896-y

**Published:** 2018-01-10

**Authors:** Charles S. Goldsmith, Sam Moon Kim, Nirmala Karunarathna, Nichole Neuendorff, L. Gerard Toussaint, David J. Earnest, Deborah Bell-Pedersen

**Affiliations:** 1 Interdisciplinary Program in Genetics, Texas A&M University, College Station TX, Texas, 77843 USA; 2 Department of Biology, Texas A&M University, College Station, Texas, TX 77843 USA; 3 Department of Neuroscience and Experimental Therapeutics, Texas A&M, Health Science Center, College of Medicine Bryan, Texas, TX 77807-3260 USA; 4 Center for Biological Clocks Research, Texas A&M University, College Station, Texas, TX 77843 USA; 5 Interdisciplinary Program in Neuroscience, Texas A&M University, College Station, Texas, TX 77843 USA

**Keywords:** Circadian clock, p38 MAPK, p38 inhibitor, Glioma, Invasiveness

## Abstract

**Background:**

The circadian clock is the basis for biological time keeping in eukaryotic organisms. The clock mechanism relies on biochemical signaling pathways to detect environmental stimuli and to regulate the expression of clock-controlled genes throughout the body. MAPK signaling pathways function in both circadian input and output pathways in mammals depending on the tissue; however, little is known about the role of p38 MAPK, an established tumor suppressor, in the mammalian circadian system. Increased expression and activity of p38 MAPK is correlated with poor prognosis in cancer, including glioblastoma multiforme; however, the toxicity of p38 MAPK inhibitors limits their clinical use. Here, we test if timed application of the specific p38 MAPK inhibitor VX-745 reduces glioma cell invasive properties in vitro.

**Methods:**

The levels and rhythmic accumulation of active phosphorylated p38 MAPK in different cell lines were determined by western blots. Rhythmic luciferase activity from clock gene luciferase reporter cells lines was used to test the effect of p38 MAPK inhibition on clock properties as determined using the damped sine fit and Levenberg–Marquardt algorithm. Nonlinear regression and Akaike’s information criteria were used to establish rhythmicity. Boyden chamber assays were used to measure glioma cell invasiveness following time-of-day-specific treatment with VX-745. Significant differences were established using *t-*tests.

**Results:**

We demonstrate the activity of p38 MAPK cycles under control of the clock in mouse fibroblast and SCN cell lines. The levels of phosphorylated p38 MAPK were significantly reduced in clock-deficient cells, indicating that the circadian clock plays an important role in activation of this pathway. Inhibition of p38 MAPK activity with VX-745 led to cell-type-specific period changes in the molecular clock. In addition, phosphorylated p38 MAPK levels were rhythmic in HA glial cells, and high and arrhythmic in invasive IM3 glioma cells. We show that inhibition of p38 MAPK activity in IM3 cells at the time of day when the levels are normally low in HA cells under control of the circadian clock, significantly reduced IM3 invasiveness.

**Conclusions:**

Glioma treatment with p38 MAPK inhibitors may be more effective and less toxic if administered at the appropriate time of the day.

**Electronic supplementary material:**

The online version of this article (10.1186/s12885-017-3896-y) contains supplementary material, which is available to authorized users.

## Background

Nearly all mammalian cells contain a 24-h molecular circadian clock. Clocks in peripheral tissues are synchronized and aligned with environmental cycles through hormonal or neuronal signals arising from the master clock in the suprachiasmatic nucleus (SCN) [1]. The SCN receives light input directly from the eye to synchronize the clock with the daily light/dark cycle [2]. In mice, the basic mechanism of the molecular clock in the SCN and peripheral tissues involves a transcription-translation feedback loop initiated by the basic helix-loop-helix (bHLH) domain-containing transcription factor BMAL1 [3–5]. During the activation phase of the cycle, BMAL1 dimerizes with either bHLH-containing CLOCK or NPAS2, and then binds to E-boxes in the promoters of the period (*Per*) and cryptochrome (*Cry*) genes. During the repression phase of the cycle, PER and CRY proteins repress the activity of BMAL/CLOCK. Following degradation of PER and CRY, newly synthesized BMAL/CLOCK complexes reinitiate the cycle the next day. In addition, BMAL/CLOCK activates the expression of ROR and Rev-erbα, which bind to ROR elements in the promoter of *Bmal1* to regulate its expression [6].

The clock mechanism is tightly linked to cell physiology and proliferation through the circadian and photic regulation of mitogen activated protein kinase (MAPK) pathway activity [7]. For example, extracellular signal-regulated kinase (ERK) MAPK activity, which promotes cell growth, differentiation, and/or mitosis, cycles in the SCN of mice under control of the circadian clock [8, 9], and ERK MAPK functions in light input to the clock [8, 10, 11]. C-Jun N-terminal kinase (JNK), involved in cell proliferation and apoptosis, also functions in mammals as an input to the clock in the SCN, and in peripheral tissues [12]. Furthermore, clock control of ERK MAPKs is conserved. In *Neurospora crassa*, clock regulation of the activity of ERK MAPK leads to the rhythmic expression of downstream clock-controlled genes [13], and in Drosophila, rhythms of ERK MAPK activity are necessary for behavioral rhythms [14].

The p38 MAPK pathway plays a major role in apoptosis, differentiation, proliferation, development, and other stress responses, and similar to ERK and JNK, evidence for a connection between the circadian clock and p38 MAPK exists. In *N. crassa*, the p38 MAPK OS-2 is rhythmically activated and functions as an output of the clock to prepare the organism for daily changes in osmotic stress [9, 15, 16]. In the chick pineal, p38 MAPK functions in circadian input to the clock [17]. Finally, p38 MAPK activity displays a circadian oscillation in the hamster SCN [9], and in 24 h light:dark cycles, rhythms of p38 MAPK activation in the chick pineal gland and mouse heart have been reported [18, 19]. However, clock-control of p38 MAPK activity in the mouse SCN cells and peripheral cells has not been investigated.

In mammals, there are four isoforms of p38 MAPK (α, β, Υ, δ) that show tissue-specific expression; however, p38α is the most ubiquitously expressed isoform [20]. The p38 MAPK pathway is stimulated by the inflammatory cytokine tumor necrosis factor-α, osmotic stress, heat shock, DNA damage, and superoxides [20]. Pathway activation (phosphorylation) leads to signaling of cell components that regulate proliferation [21] and apoptosis [22]. As such, p38 MAPK, particularly p38α, can function as a tumor suppressor [23–25]. Because increased expression and activity of p38 MAPK also correlates with poor prognosis in several types of cancers [26, 27], including glioblastoma [28–30], p38 MAPK inhibitors have attracted significant attention for use in chemotherapy [24]. Yet despite their potential, high toxicity and off-target effects have severely limited their therapeutic value [31]. Furthermore, the prospective for time-of-day treatment effects, based on clock-control of p38 MAPK activity in normal cells versus increased activity in certain cancers, as a way to increase efficacy and reduce toxicity of the inhibitors has not been explored.

To address these outstanding questions, we examined the levels and rhythmicity of phosphorylated p38 MAPK in neural SCN and glial cells, and in peripheral fibroblasts cells. We show that p38 MAPK is rhythmically activated in all 3 cell types, and that rhythmicity and p38 MAPK levels are dependent on a functional circadian oscillator in SCN and fibroblast cells. Although the molecular clock was functional in highly invasive rat glioblastoma IM3 cells, the levels of phosphorylated p38 MAPK were high and arrhythmic. Despite the lack of rhythmicity in p38 MAPK phosphorylation, inhibition of p38 MAPK activity with VX-745 led to a time-of-day-specific reduction in the invasive properties of IM3 cells corresponding to the time when phosphorylated p38 MAPK levels are low in glial cells. Together, these data support the idea that p38 MAPK inhibitors may be more effective and less toxic if given at the appropriate times of day.

## Methods

### Cell lines and culture conditions

Experimental analyses were performed in vitro using the following cell lines: mouse *Per2*^*Luc*^ SCN cells and fibroblasts, mouse *Bmal1-dLuc* fibroblasts, mouse *Per1*^*ldc*^/*Per2*^*ldc*^ SCN cells and fibroblasts, human astroglia (HA), and C6 and IM3 rat glioma cells. SCN cell lines were derived from fetal SCN of *mPer2*^*Luc*^ [32] and wild type (129/SV) or *Per1*^*ldc*^*/ Per2*^*ldc*^ mice [33], and immortalized with the adenovirus E1A gene [34]. Fibroblast cell lines were derived from the skin of *mPer2*^*Luc*^, wild type (129/SV) or *Per1*^*ldc*^*/ Per2*^*ldc*^ neonatal mice fibroblasts and isolated fibroblasts were immortalized with the adenovirus E1A gene. Mouse *Bmal1-dLuc* fibroblasts were provided by Dr. Andrew Liu (University of Memphis, Memphis, TN [35]. The rat glioma cell line C6 was obtained from the American Type Culture Collection (ATCC, Manassas, VA, USA) and the invasive IM3 line was derived from a sub-population of parental cells isolated through three successive selection procedures requiring their invasion to the bottom chamber of a Boyden-type manifold [36]. The HA line (Human Astrocytes #1800) was obtained from ScienCell Research Laboratories (Carlsbad, CA, USA) and consists of human astrocytes isolated from the cerebral cortex. HA cells, which are guaranteed to passage 10, were used at passage 4–6, and were certified to be free of biological contaminants.

SCN cell lines were maintained on laminin-coated 60 mm cell culture dishes (Corning, Corning, NY) in Minimum Essential Medium (MEM; Invitrogen, Carlsbad, CA, #10370–021) supplemented with 10% fetal bovine serum (FBS; Hyclone, Thermo Fischer, Waltham, MA), glucose (3000 μg/mL), and L-glutamine (292 μg/mL). Fibroblasts were grown on 60 mm culture dishes in Dulbeco’s Modified Eagle Medium (DMEM; Invitrogen) containing 10% FBS (Hyclone), L-glutamine (292 μg/mL) and glucose (4500 μg/mL). The C6 and IM3 glioma cell lines were similarly cultured on 60 mm dishes in DMEM supplemented with 2% FBS and equivalent concentrations of L-glutamine and glucose. The HA astrocyte line was maintained in 60 mm dishes containing Astrocyte Medium (AM; ScienCell Research Laboratories, Carlsbad, CA), 2% FBS and growth (AGS)/antibiotic (penicillin/streptomycin) supplements. All cultures were maintained 37 °C and 5% CO_2_, and passaged every 2–3 days at a 1:3 ratio. To synchronize SCN, fibroblast, HA, C6 and IM3 cells, cultures were serum shocked as described [37] with medium containing 50% horse serum. During time course analyses, *Per2*^*Luc*^ and *Per1*^*ldc*^/*Per2*^*ldc*^ cell lines (both SCN cells and fibroblasts) were cultured in serum-free growth media, whereas HA, C6 and IM3 cells were maintained in growth medium containing 1% FBS and then harvested by trypsinization (0.05% Trypsin/EDTA (Invitrogen #15400) at 4 h intervals for 48 h. After trypsin inactivation with 10% FBS (Hyclone, Thermo Fisher Scientific, Waltham, MA), cells were pelleted by centrifugation, immediately flash frozen in liquid nitrogen and stored at −80 °C until subsequent analysis.

### Immunoblotting

To extract protein for western blotting, 250 μl of extraction buffer (20 mM Tris pH 7.5; 137 mM NaCl; 1% Triton X-100; 10% glycerol; 10 mM NaF; 10 mM β-glycero-phosphate; 2 mM EDTA; 1 mM PMSF; 1 mM sodium ortho-vanadate; 1× HALT Protease Inhibitor Cocktail (Thermo Scientific, Rockford, IL)) was added to cell pellets on ice. The pellets were sonicated using a Branson Sonifier 450 equipped with a microtip for 10 s at 10% amplitude. Samples were then placed on ice for 15 min before pelleting cell debris at max rpm for 5 min at 4 °C. An aliquot of protein extract was removed, and protein amounts were quantitated using the *DC* Protein Assay (Bio-Rad; Hercules, CA). Protein (30 μg) was boiled for 5 min with 1× Laemmli buffer before being separated via 10% SDS-PAGE. Protein was transferred from gels to Immobolin-P PVDF membrane (EMD Millipore, Billerica, MA) and immunoblotted according to antibody protocols. For detection of phospho-p38 MAPK (α, β forms), membranes were probed with mouse anti-phospho-p38 primary (#9216 Cell Signaling, Beverly, MA), and anti-mouse-HRP secondary (#170–6516 BioRad, Hercules, CA) antibodies. For detection of total p38 MAPK (α, β, Υ forms) membranes were probed with rabbit anti-p38 primary (#9212 Cell Signaling, Beverly, MA), and anti-rabbit-HRP secondary (#170–6515 BioRad, Hercules, CA) antibodies. For detection of actin, membranes were probed with mouse anti-actin primary (#A4700 Sigma-Aldrich, St Louis, MO). Blots were visualized on X-ray film (Phenix, Candler, NC) with Super Signal West Fempto Chemiluminescent Substrate (Thermo Scientific, Rockford, IL).

### Real-time analysis of mPER2::LUC bioluminescence

Analysis of bioluminescence from *mPer2*^*Luc*^ SCN cells and *Bmal1-dLuc* fibroblasts was performed as described previously [34, 35]. SCN *mPer2*^*Luc*^ cultures were placed in DMEM recording media (Sigma-Aldrich, St. Louis, MO) containing 10 mM Hepes, 0.03% NaHCO3, N2 supplement (1X; Invitrogen), 4.510 g/L glucose, 25 units/ml penicillin, 25 μg/ml streptomycin and 0.1 mM beetle luciferin (Promega, Madison, WI). Bioluminescence recording from *Bmal1-dLuc* fibroblast cultures was performed in DMEM recording medium containing 1 μM forskolin, 25 mM HEPES, 292 μg/ml L-glutamine, 100 units/ml penicillin, 100 μg/ml streptomycin and 0.1 mM beetle luciferin (Promega). Individual cultures were sealed airtight with sterile glass coverslips (VWR, Radnor, PA), and sterile silicon grease (Dow Corning, Midland, MI). The temporal patterns of mPER2::LUC and *Bmal1-dLuc* bioluminescence were analyzed using an automated 32-channel luminometer (LumiCycle; Actimetrics, Wilmette, IL, USA) that was maintained within a standard cell culture incubator at 35 °C. Bioluminescence from individual cultures was continuously recorded with a photomultiplier tube (PMT) for ~70 s at intervals of 10 min for 6–8 days. Due to the transient induction of bioluminescence following the medium change at the initiation of this analysis, the first cycle was excluded from data analysis. Bioluminescence data was analyzed using the Lumicycle Analysis program (Actimetrics). For each raw data set, baseline drift was removed by fitting a polynomial curve with an order equal to one less than the number of recorded cycles. Rhythm parameters were determined from baseline-subtracted data the damped sine fit and Levenberg–Marquardt algorithm.

### RNA extraction and real-time PCR

Total cellular RNA was extracted from individual cultures of HA astroglia, C6 glioma and IM3 glioma using miRNeasy kit (Qiagen, Inc., Valencia, CA) according to the manufacturer’s protocols. Total RNA was estimated using a Nanodrop ND2000 (Thermo Scientific, Rockford, IL). Relative quantification of *Bmal1* and *Per2* mRNA abundance in all samples was performed using SYBR-Green real-time PCR technology (ABI) as described previously [38, 39]. To generate single-strand cDNAs, total RNA (1 μg) from individual samples was reverse transcribed using random hexamers and Superscript III reverse transcriptase kit (Invitrogen). Real-time PCR analysis was performed on duplicate aliquots using the cDNA equivalent of 1 ng of total RNA for each sample. The PCR cycling conditions were: 1) serial heating at 50 °C for 2 min and 95 °C for 10 min, 2) amplification over 40 cycles at 95 °C for 15 s and 60 °C for 1 min, and 3) dissociation at 95 °C for 15 s, 60 °C for 1 min, 95 °C for 15 s and 60 °C for 15 s. To control for differences in sample RNA content, cyclophilin A (*Ppia*) was amplified with the cDNA equivalent of 1 ng total RNA from the same samples. Consistent with our previous studies using this gene in a similar manner [39, 40], *Ppia* showed no sign of circadian or ultradian variation. The comparative CT method was utilized to calculate the relative abundance for a given clock gene mRNA by normalization to corresponding *Ppia* levels in each sample and to a calibrator consisting of pooled cDNA from multiple samples.

The following probes and primers were designed using PrimerExpress software (ABI):

*mBmal1* forward: 5′- CCAAGAAAGTATGGACACAGACAAA -3′;

*mBmal1* reverse: 5′- GCATTCTTGATCCTTCCTTGGT -3′;

*rBmal1* forward*: 5’-*GCAATCTGAGCTGCCTCGTT*-3’*

*rBmal1* reverse*: 5’-*CCCGTATTTCCCCGTTCACT*-3’*

*mPer2* forward*: 5’-*ATGCTCGCCATCCACAAGA*-3’*

*mPer2* reverse*: 5′ -*GCGGAATCGAATGGGAGAAT*- 3’*

*rPer2* forward*: 5’-*CCCATCCCACACTTGCCTC*-3’*

*rPer2* reverse*: 5’-*CACTGTGCCAGCCGGG*-3’*

*Ppia* forward: 5′- TGTGCCAGGGTGGTGACTT -3′;

*Ppia* reverse: 5′- TCAAATTTCTCTCCGTAGATGGACTT -3′

### Glioma invasion assay

C6 and IM3 rat glioma cells were propagated on 60 mm dishes as described earlier and split 1:3 every 2 days. Approximately 24 h after plating, all cultures were exposed for 2 h to medium containing 50% horse serum to facilitate circadian oscillation synchronization across cultures. To examine the time-dependent effects of p38 MAPK inhibition on the invasive phenotype of glioma cells, IM3 cultures were treated with vehicle (DMSO) or 20 μM VX-745 (Tocris Bioscience, Bristol, UK) for 6 h such that the mid-point of the treatment interval occurred at 12 h or 24 h after serum shock (so as to respectively coincide with the peak and trough of p38 MAPK phosphorylation in HA cultures). Cultures of untreated parental C6 glioma were collected at similar times and analyzed in parallel to establish comparative differences in invasive phenotype. Cell invasion properties were determined by plating suspensions of 150,000 C6 (untreated) or IM3 (DMSO- or VX-745-treated) glioma cells/ml in serum-free DMEM (500 μl total volume) in the upper chamber of a Matrigel-coated membrane insert (24-well insert; pore size, 8 μm; BD Biosciences, San Jose, CA). DMEM medium containing 10% FBS in the lower chamber served as the chemoattractant. Cells were subsequently incubated at 37 °C for 8 h based on previous optimization of assay conditions [36]. Non-invading cells were removed with cotton swabs. Those cells that had migrated to the lower side of the membrane were fixed and stained 1% toluidine blue. For all C6 and IM3 cultures, invading cells were quantitated on eight different membranes by counting across a diameter of each membrane under 20X microscopic magnification. Statistical comparisons of C6, DMSO IM3 and VX-745 IM3 groups within each time point were established by normalizing invasion cell counts in each culture to the averages of time-matched C6 cells.

### Statistical methods

Statistical analysis of rhythmic data from western blots was performed as described [15]. Briefly, using Prism software (GraphPad Software, San Diego, CA), comparison of rhythmic data to either a line or sine wave with nonlinear regression and Akaike’s information criteria established if a rhythm was significantly (*p* < 0.05) more similar to a sine wave than a line. The error bars in graphs represent standard error of the mean (SEM) of at least three biological replicates. Independent pooled *t*-tests were performed to determine the significance of cell-specific differences and treatment-induced changes in cell invasion. In each case, differences were considered significant at *p* < 0.05.

## Results

**Fig. 1:**
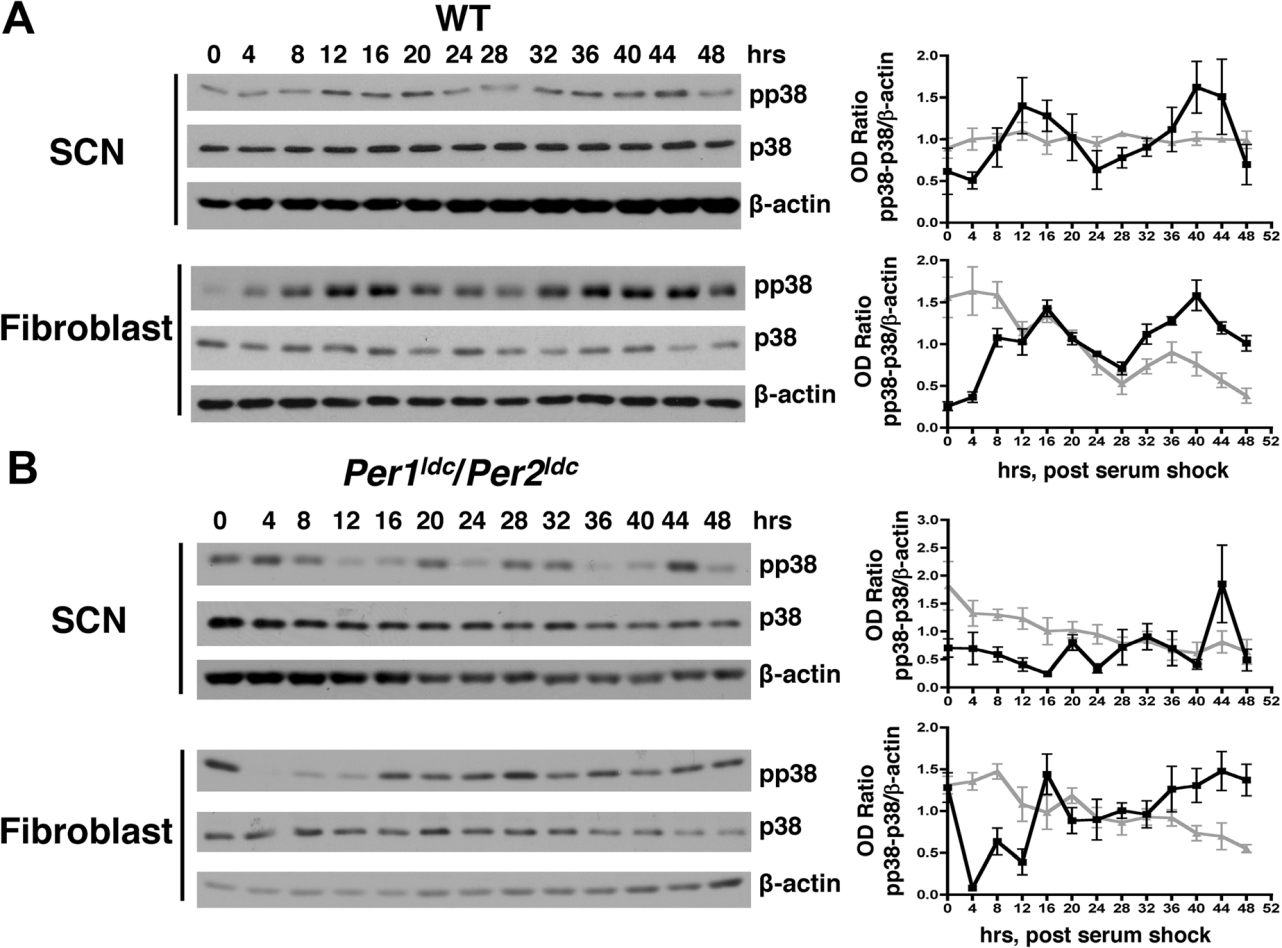
p38 MAPK activity is rhythmic in SCN and fibroblast cultures. **a** Representative western blots and densitometric analyses of p38 MAPK activation in WT mouse SCN (*n* = 3) and fibroblast (*n* = 4) cultures harvested following serum shock at 4-h intervals for 2 days. Western blots depict immunoreactive signal for phospho-p38 MAPK (pp38), total p38 MAPK (p38) and β-actin in the same samples. Graphs on the right depict the average immunoreactive signal (pp38 or p38) normalized to β-actin obtained from 3 to 4 independent sets of samples. The pp38 MAPK (black squares) signal was rhythmic in SCN cells and fibroblasts as confirmed by statistical best fit to a sine wave (*p* < 0.05; *n* = 3 for SCN cells, and n = 4 for fibroblasts, ± SEM). Total p38 protein (gray triangles) was arrhythmic in SCN and fibroblast cultures as confirmed by statistical best fit to a line (p < 0.05; n = 3 for SCN cells, and n = 4 for fibroblasts, ± SEM). **b** Representative western blots and densitometry examining p38 MAPK activation in clock-disrupted *Per1*^*ldc*^/*Per2*^*ldc*^ SCN (*n* = 3) and fibroblast (*n* = 5) cultures as described in A. The ratios of pp38/β-actin (black circles) and p38/β-actin (gray triangles) signal were arrhythmic in SCN cells and fibroblasts as confirmed by statistical best fit to a line (*p* < 0.05; n = 3 for SCN cells, and n = 5 for fibroblasts, ± SEM). Pictures of full gels are shown in Additional file 1

To determine if p38 MAPK activity rhythms are conserved in higher eukaryotes, we used immortalized murine cell lines as an in vitro model. Cell lines derived from the SCN and serum-shocked fibroblasts were harvested every 4 h over two days. Relative levels of phosphorylated p38 MAPK, but not total p38 protein, were marked by circadian variation in both SCN and fibroblast cultures. In SCN cultures, phospho-p38 MAPK levels oscillated with a period of 25.5 ± 0.9 h and peak levels at 12 h and 40 h after serum shock (Fig. 1a). In contrast, levels of total p38 protein were arrhythmic in SCN cells (Fig. 1a). Fibroblasts also exhibited rhythmic phospho-p38 MAPK levels with a period of 25.6 ± 0.9 h and peaks at 16 h and 40 h after serum shock (Fig. 1a). In the same fibroblast cultures, the levels of total p38 MAPK protein were variable, but arrhythmic over the time course for sampling (Fig. 1a). Consistent with circadian regulation of the p38 MAPK pathway, the levels of phospho-p38 MAPK were variable and arrhythmic in both SCN and fibroblast cell lines derived from clock-disrupted *Per1*^*ldc*^*/ Per2*^*ldc*^ mutant mice (Fig. 1b). These data demonstrated that phosphorylation and activation of p38 MAPK is clock-controlled in SCN and fibroblast cell cultures.

**Fig. 2:**
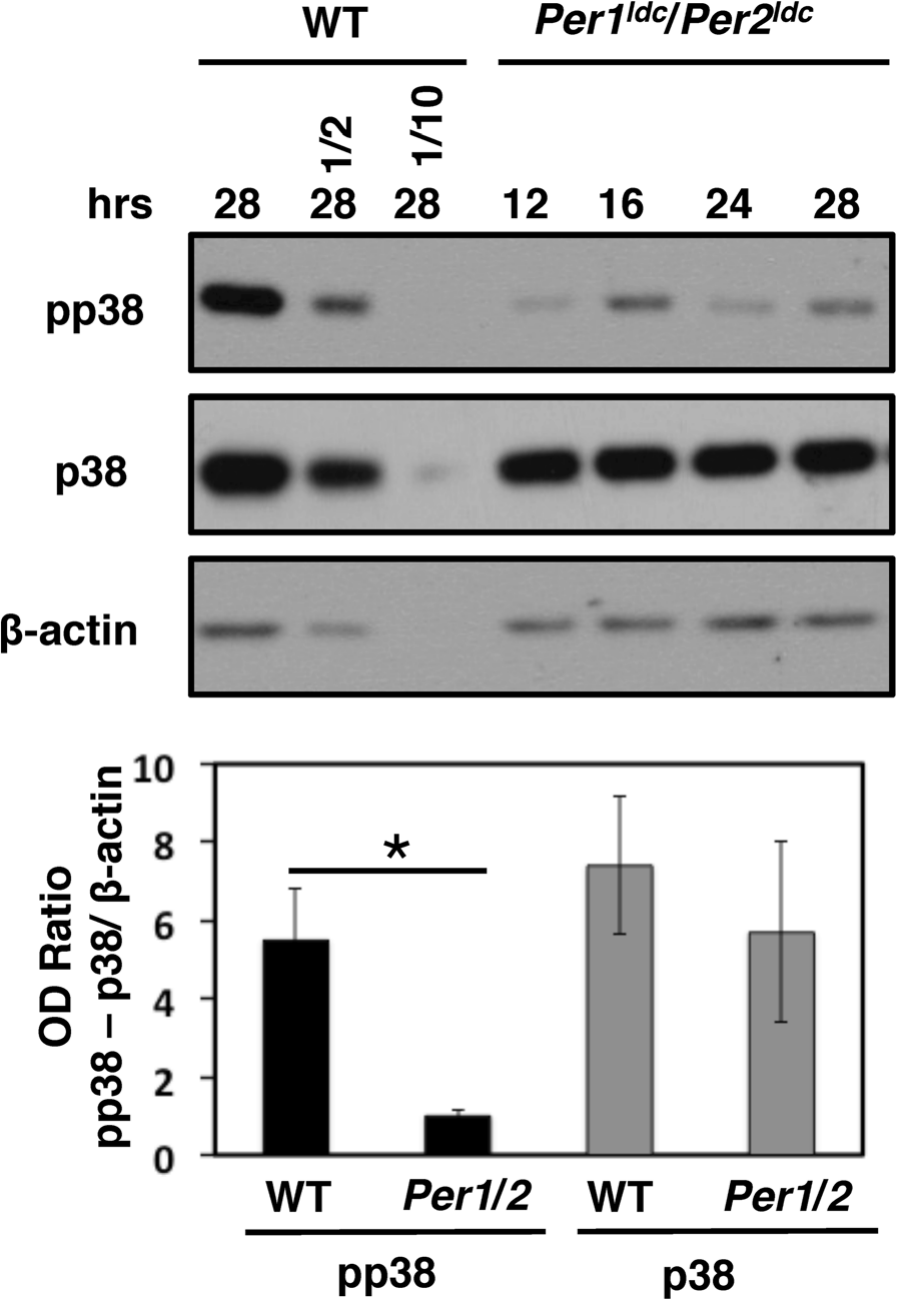
p38 MAPK activity is low in clock-disrupted *Per1*^*ldc*^/*Per2*^*ldc*^ mutant fibroblasts. Representative western blots of protein extracts from WT and *Per1*^*ldc*^/*Per2*^*ldc*^ fibroblasts (from Fig. 1) harvested at the indicated hour after serum shock probed with anti-phospho-p38 (pp38), anti-total p38 (p38) antibodies, and control anti-β-actin antibodies. A ½ and 1/10th fraction of WT protein harvested at 28 h was loaded as a reference. The data are plotted below (n = 3; ± SEM). The asterisk denotes a significant decrease in pp38 MAPK levels (*p* < 0.05)

To examine if the levels of phosphorylated p38 MAPK are altered in cells lacking a functional clock, phosphorylated p38 MAPK and total p38 MAPK levels were analyzed in WT and *Per1*^*ldc*^*/ Per2*^*ldc*^ fibroblasts. Compared to WT phosphorylated p38 MAPK levels observed at 28 h post-serum shock, the time of day when phospho-p38 MAPK is at its rhythmic trough (Fig. 1a), phosphorylated p38 MAPK levels in the *Per1*^*ldc*^*/ Per2*^*ldc*^ mutant fibroblasts were significantly lower (Fig. 2). Similar low levels were observed in mutant fibroblasts at all times tested (Fig. 2). Alternatively, total p38 MAPK levels were similar in WT and *Per1*^*ldc*^*/ Per2*^*ldc*^ fibroblasts at all times of day examined. These data revealed that functional *Per1* and *Per2* are required for normal regulation of p38 MAPK phosphorylation, and supported clock activation of p38 MAPK.

**Fig. 3:**
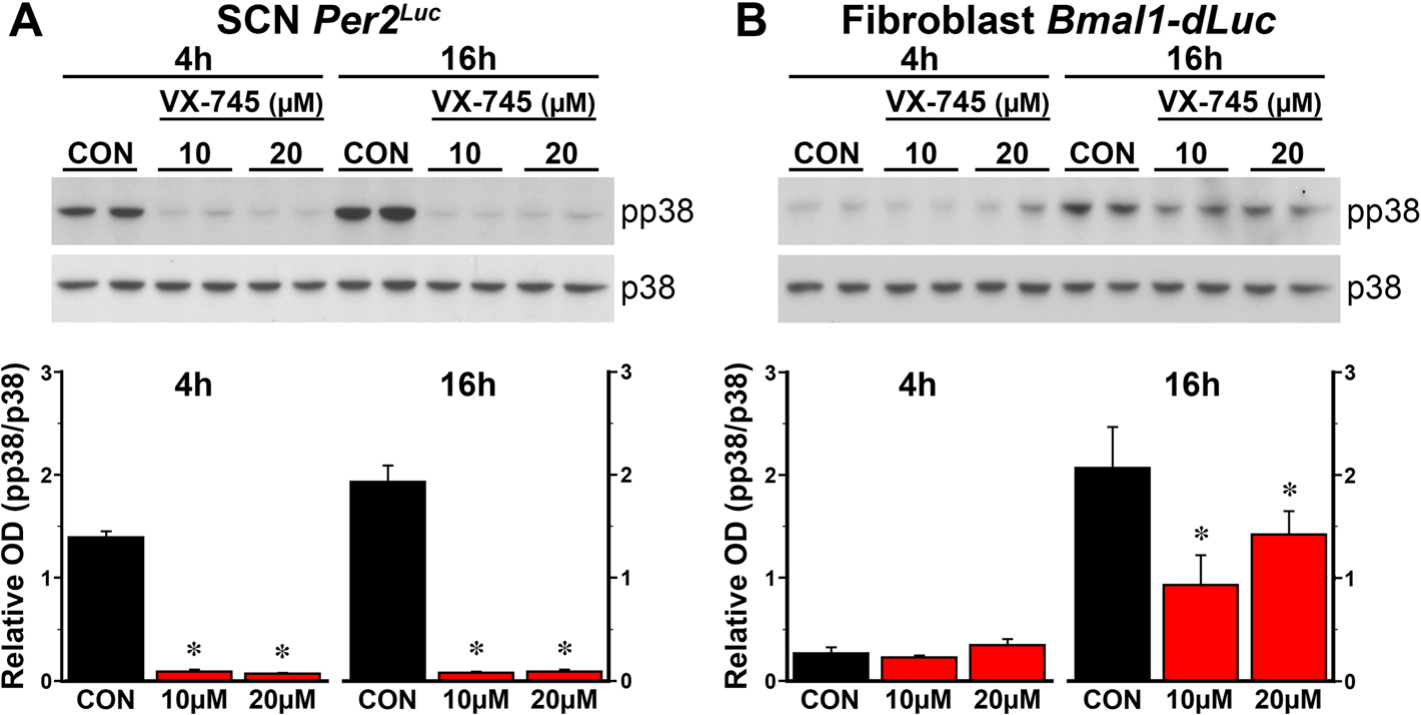
VX-745 inhibits p38 MAPK activity in cultured *Per2*^*Luc*^ SCN cells and *Bmal1-dLuc* fibroblasts. Representative western blots (top) and densitometric analyses (bottom) of p38 MAPK activation in *Per2*^*Luc*^ SCN (**a**) and *Bmal1-dLuc* fibroblast (**b**) cultures treated for 1 h with DMSO (CON) (n = 4) or VX-745-treated (10 μM or 20 μM; n = 4) at 4 h or 16 h post-serum shock. Bar graphs depict the ratios of pp38/p38 MAPK immunoreactive signal. Asterisks denote treatment times in which p38 MAPK phosphorylation in VX-745-treated SCN cells or fibroblasts were significantly decreased (*p* < 0.05) compared to control cultures. OD = optical density

In previous studies, application of p38 MAPK inhibitors SB203580 or SB202190 to U20S human osteosarcoma cells, and rat C6 glioblastoma cells, led to period lengthening of clock gene rhythms, suggesting that p38 MAPK modulates the activity of core clock components [41]. However, both of these inhibitors have off-target effects, including inhibition of CKIε previously shown to modulate the activity of components of the circadian oscillator [42–44]. Thus, to determine if the activity of p38 MAPK modulates circadian timekeeping function of both SCN and peripheral clocks, we examined the effects of VX-745, a potent and highly specific p38 MAPK inhibitor [45, 46], on p38 MAPK phosphorylation and on clock gene oscillations in mouse *Per2*^*Luc*^ SCN cells and *Bmal1-dLuc* fibroblasts. First, VX-745 inhibition of p38 MAPK phosphorylation was examined near the peak (hour 16) and trough (hour 4) of its activity (using two different doses: 10 μM and 20 μM) (Fig. 3). Consistent with the rhythmic time course data (Fig. 1), the levels of phospho-p38 MAPK, but not total p38 MAPK, were higher at hour 16 as compared to hour 4 (Fig. 3). However, the fold change in phospho-p38 MAPK levels was greater in *Bmal1-dLuc* fibroblasts (5X) than in *Per2*^*Luc*^ SCN (1.5X) cells. Treatment of *Per2*^*Luc*^ SCN cultures with 10 μM or 20 μM VX-745 at hour 4 or 16 had no effect on the total levels of p38 MAPK, but led to a significant reduction (>83%) in phospho-p38 MAPK levels relative to time-matched controls (Fig. 3a). In *Bmal1-dLuc* fibroblasts, 10 μM and 20 μM VX-745 also had no effect on total levels of p38 MAPK, but led to significant inhibition of p38 MAPK phosphorylation when treatment occurred at hour 16. At hour 4, phospho-p38 MAPK levels were low, and no further reduction occurred upon treatment with 10 or 20 μM VX-745 (Fig. 3b).

**Fig. 4:**
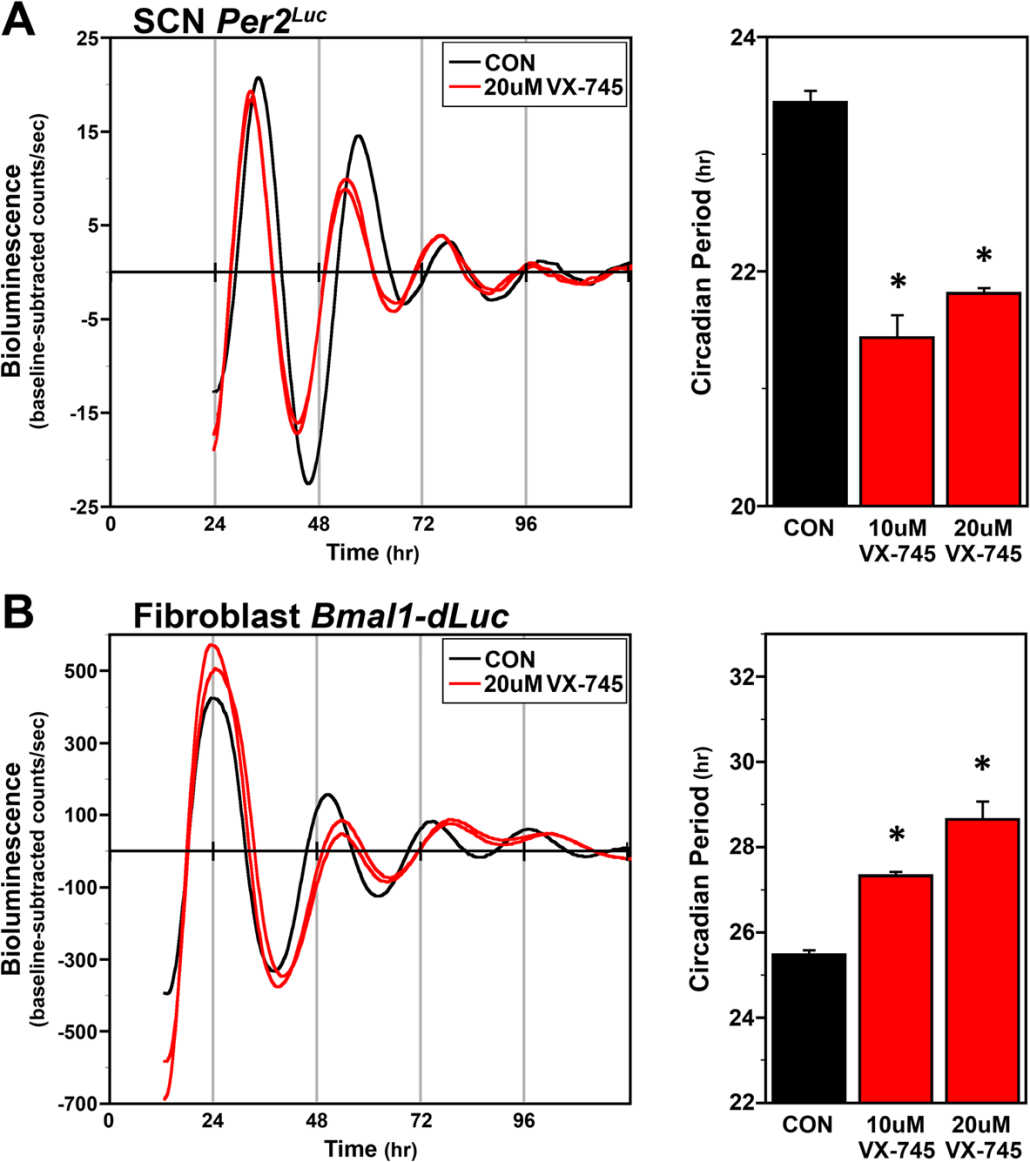
VX-745 shortens the period of clock gene rhythms in cultured *Per2*^*Luc*^ SCN cells and *Bmal1-dLuc* fibroblasts. The left panels depict individual recordings of ensemble bioluminescence (expressed as detrended baseline-subtracted counts per second) from representative cultures of serum-shocked *Per2*^*Luc*^ SCN cells (**a**) and *Bmal1-dLuc* fibroblasts (**b**) treated with DMSO (CON) or 20 μM VX-745. Right panels show bar graph comparisons of the circadian period (mean + SEM) of ensemble clock gene rhythms in control (CON) (*n* = 6) and VX-745-treated (10 μM or 20 μM; *n* = 6) SCN cells and fibroblasts. Asterisks indicate that the period of the SCN PER2::LUC and fibroblast *Bmal1-dLuc* rhythms in VX-745-treated cultures was significantly different (*p* < 0.05) from that in DMSO controls

To determine if p38 MAPK activity modulates circadian timekeeping, we assayed bioluminescence rhythms in *Per2*^*Luc*^ SCN or *Bmal1-dLuc* fibroblast cultures during treatment with vehicle (DMSO) or VX-745 (Fig. 4). All DMSO-treated *Per2*^*Luc*^ SCN and *Bmal1-dLuc* fibroblast cultures exhibited circadian rhythms of bioluminescence that persisted for at least 3–4 cycles with circadian periods of 23.4 h and 25.5 h, respectively. The peak of SCN PER2::LUC expression occurred at ~36 h after the initiation of bioluminescence analysis, and the peak of *Bmal1-dLuc* in fibroblasts occurred at ~24 h following serum shock, similar to published data [34, 47]. The period difference observed between the PER2::LUC rhythms in SCN cells (Fig. 4a) and *Bmal1-dluc* oscillations in fibroblasts is consistent with our published data [34, 47] and reflects differences in cell types and clock gene reporters. In *Per2*^*Luc*^ SCN cells or *Bmal1-dLuc* fibroblasts treated with 10 μM or 20 μM VX-745, rhythms of bioluminescence persisted for 3–4 cycles similar to the control. However, VX-745 had a modulatory effect on circadian period in both SCN PER2::LUC and fibroblast *Bmal1-dLuc* rhythms. In SCN cultures, 10 μM and 20 μM VX-745 treatment significantly decreased the period of the rhythm in PER2::LUC expression relative to that observed in DMSO controls (Fig. 4a). The period of the PER2::LUC bioluminescence rhythms in VX-745-treated SCN cultures was decreased by 1.9 h with 10 μM, and by 1.6 h with 20 μM. VX-745 had the opposite effect on *Bmal1-dLuc* rhythms in fibroblast cultures, and significantly increased the period of *Bmal1-dLuc* rhythms by 1.9 h (10 μM) and by 2.2 h (20 μM) in comparison to DMSO controls (Fig. 4b). These data support that p38 MAPK has cell type-specific effects on the molecular clock.

**Fig. 5:**
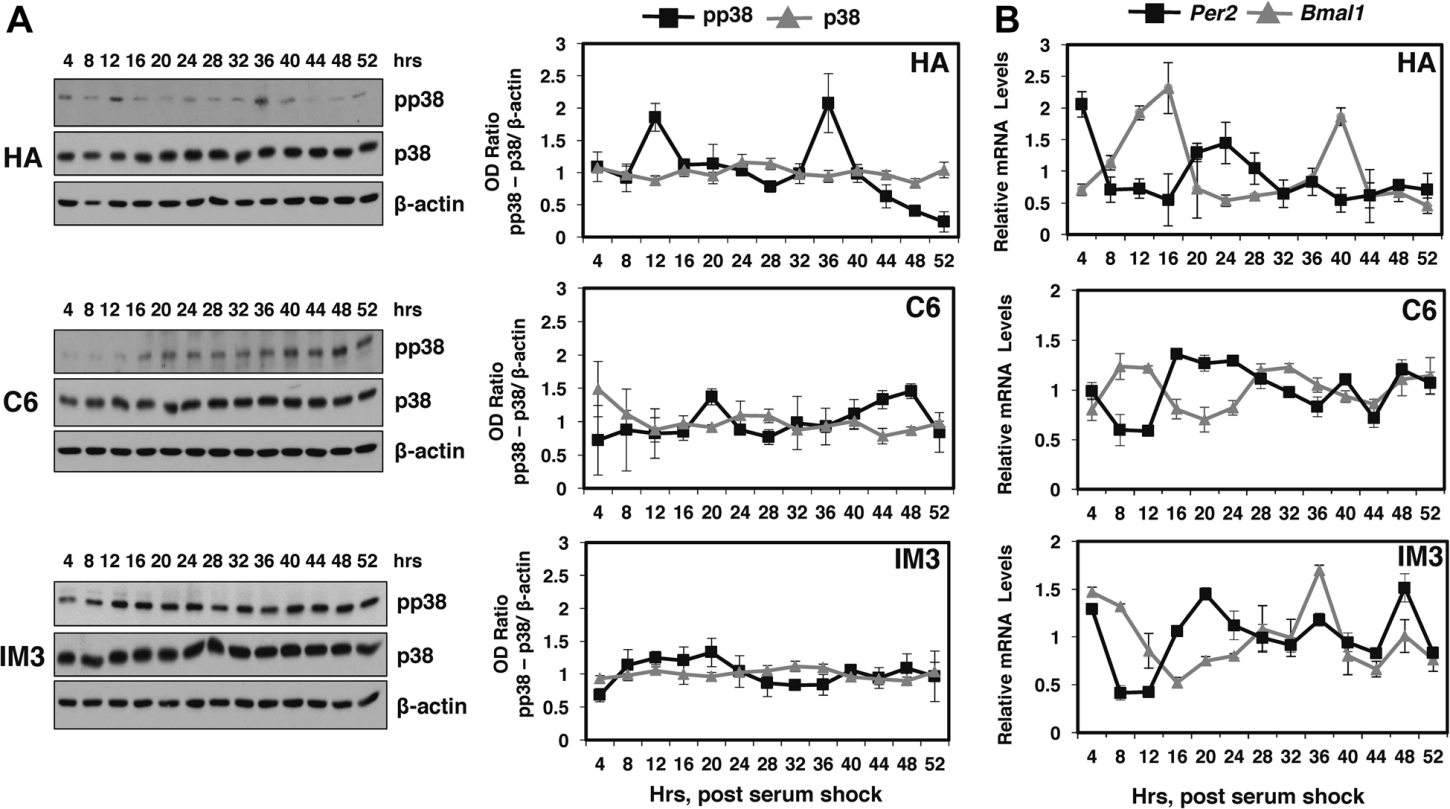
Temporal profiles of p38 MAPK activity in astroglial and glioma cell lines. **a** Representative western blots (left) and densitometric analyses (right) of p38 MAPK activation in astroglial (HA) and C6 and IM3 glioma cultures harvested following serum shock at 4-h intervals for 2 days as described in Fig. 1. Graphs on the right depict the average immunoreactive signal of phospho-p38 MAPK (pp38) or total p38 MAPK (p38) normalized to β-actin obtained from 3 independent sets of samples. The pp38 MAPK (black squares) signal was rhythmic in HA cells as confirmed by statistical best fit to a sine wave (*p* < 0.05; ± SEM). Ratios of pp38/β-actin in C6 and IM3 glioma cultures and of p38/β-actin (gray triangles) signal in all 3 cell lines were arrhythmic as confirmed by statistical best fit to a line (*p* < 0.05; ± SEM). **b** Real-time PCR determinations (mean ± SEM) of the relative levels of *Bmal1* and *Per2* mRNA in HA, C6, and IM3 cultures (n = 3) harvested at 4-h intervals for 2 days as described in Fig. 1. The plotted values correspond to the ratios of *Bmal1* or *Per2* mRNA signal normalized to *Ppia* mRNA levels in which the maximal value for each gene was set at 100%. *Bmal1* and *Per2* mRNA levels were rhythmic in the 3 cell types as confirmed by statistical best fit to a sine wave (*p* < 0.05; n = 3, ± SEM). Pictures of full gels are shown in Additional file 2

The p38 MAPK plays a role in the invasive phenotype of glioblastoma multiforme [48]. Therefore, we speculated that clock control of p38 MAPK levels may be altered in glioma cells as compared to normal glial cells, and that loss of clock control of p38 MAPK may influence the invasive phenotype. To test this idea, we compared circadian clock control of p38 MAPK activity in control astroglial (HA) cells to non-invasive rat C6 glioma cells and highly invasive IM3 glioma cells. The levels of phosphorylated p38 MAPK fluctuated rhythmically with a period of 22.7 ± 1.2 h**,** and with peak activity at 12 and 36 h after serum shock (Fig. 5a). In contrast to the clear oscillatory regulation of p38 MAPK activity in HA cells, in parental C6 and highly invasive IM3 glioma cells, phospho-p38 MAPK levels fluctuated, but were arrhythmic over the time course. Consistent with the temporal profile of total p38 MAPK in SCN and fibroblast cell lines (Fig. 1), the levels total p38 protein were constant in HA, C6 and IM3 cultures (Fig. 5a). The levels of *Bmal1* and *Per2* mRNA were rhythmic in all 3 cell lines as judged by statistical best fit to a sine wave. The period of *Per2* mRNA rhythms was 26.4±1.6 in HA, 31.3±2.0 in C6, and 24.1± 1.4 in IM3 cells, and the period of *Bmal1* mRNA rhythms was 24.5±1.2 in HA, 20.7 ±0.8 in C6, and 29.3±1.6 in IM3 cells. *Per2* mRNA expression peaked ~20–24 h after serum shock (Fig. 5b), whereas peak *Bmal1* mRNA levels occurred at 8–16 and 36–40 h after serum shock. Importantly, the observed phase difference between the *Bmal1* and *Per2* mRNA rhythms in HA, C6 and IM3 cultures is consistent with previous evidence for antiphase relationship between these clock gene rhythms in cultured rodent SCN cells and fibroblasts [34, 38, 49, 50]. Although there was some variation in period in clock gene rhythms between the different cell types, these data indicated that a functional molecular clock is present and synchronized by serum shock in all 3 cell types maintained under these culture conditions.

**Fig. 6:**
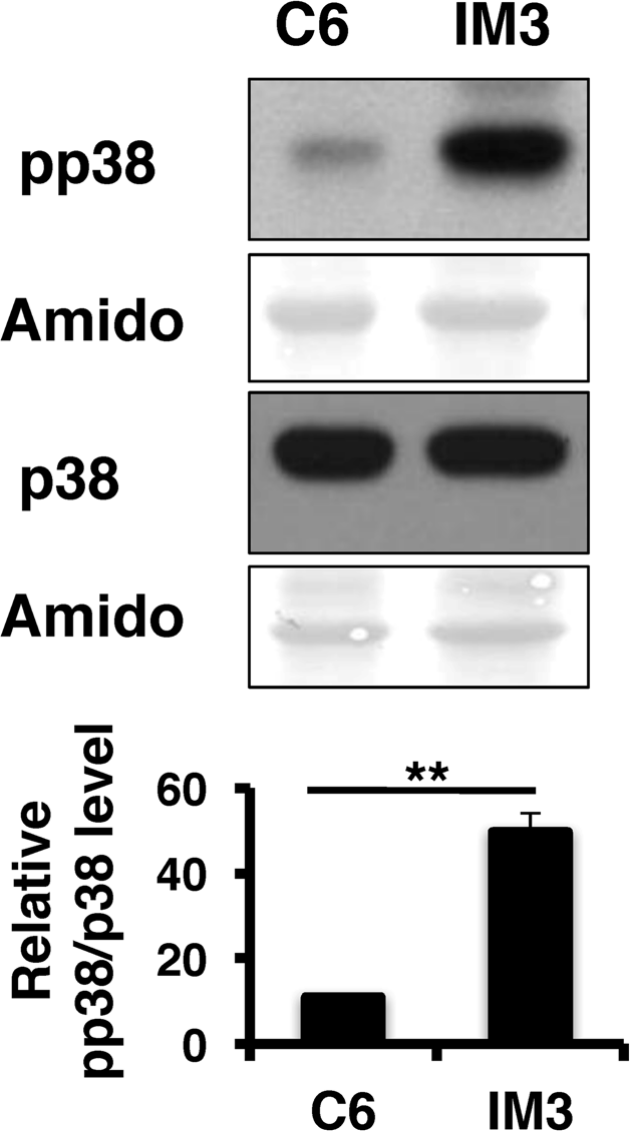
p38 MAPK activity differs among glial cells with normal or malignant phenotypes. Representative western blots of protein isolated from the indicated cells lines harvested 24 h after serum shock and probed with anti-phospho-p38 (pp38), or anti-total p38 (p38) antibodies. Amido-stained membranes were used to normalize protein loading. The graph below shows the average pp38 MAPK signal normalized to total p38 MAPK levels (n = 3, ± SEM). pp38 MAPK levels were significantly lower in C6 cells compared to IM3 (**p < 0.05) cells

In the time course experiments, the levels of phospho-p38 MAPK appeared high in IM3 cells and low in C6 cells (Fig. 5a). To better determine if phospho-p38 MAPK levels differed in accord with their invasive phenotype, the 24 h samples from the time courses were separated on the same gel and probed with antibodies to detect phospho-p38 MAPK or total p38 MAPK (Fig. 6). Compared to non-invasive C6 cells, phospho-p38 MAPK levels increased by ~5-fold in IM3 cells, while total levels of p38 MAPK were similar in both cell lines. These data are consistent with p38 MAPK activity impacting cell invasion [48].

**Fig. 7:**
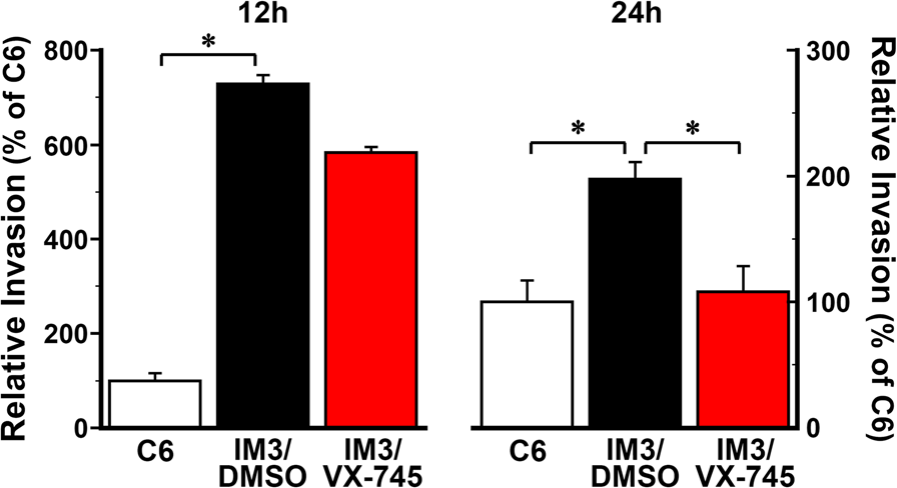
Time-dependent inhibition of invasiveness of IM3 glioma cells by VX-745. Boyden chamber invasion assays were performed on untreated parental C6 cultures and IM3 glioblastoma cultures that were treated for 6 h with DMSO or 20 μM VX-745 at two different times: 12 h or 24 h after serum shock. Plotted values correspond to the ratio of invasion cell counts in each culture adjusted relative to the averages of time-matched C6 cells, which were set at 100%. Data are represented as means (±SEM; *n* = 8). Relative cell invasion in DMSO-treated IM3 glioma cultures was significantly greater at both time points than that observed in parental C6 cells, and in IM3 glioma cultures treated with VX-745 at 24 h after serum shock (*, T-test *p* < 0.05)

We next wanted to determine if p38 MAPK inhibitors would show a time of day effect on invasiveness. As expected, in IM3 cells treated with DMSO at 12 or 24 h after serum shock, cell invasion was significantly increased (*p* < 0.05) by 2–7 fold relative to time-matched untreated C6 cultures, and IM3 invasiveness was significantly inhibited by VX-745 at 24 h post serum shock (p < 0.05) (Fig. 7), the time when phosphorylated p38 MAPK levels are lowest in control HA cells (Fig. 6). Moreover, VX-745 treatment at 24 h after serum shock effectively suppressed the distinctive invasive phenotype of IM3 glioma cultures, such that invading cell counts were equivalent to those observed in untreated parental C6 cells. The effects of p38 MAPK inhibition were time-of-day-dependent, as IM3 invasiveness was not significantly reduced when the cells were treated with VX-745 at 12 h post serum shock (Fig. 7), the time when phosphorylated p38 MAPK levels are at their peak in HA cells (Fig. 6). These data raised the intriguing possibility that targeted inhibition of p38 MAPK in glioblastoma might reduce brain invasion, and decrease toxicity to normal cells, if administered at times of day when phosphorylated p38 MAPK levels are low in normal cells under control of the circadian clock.

## Discussion

Our studies support that rhythms in p38 MAPK activation are conserved in diverse organisms and cell types. Here, we show that mouse SCN and fibroblast cell lines, have an endogenous rhythm in activation that is dependent on a functional circadian oscillator. Confirmation of clock regulation came from demonstrating that the rhythms in p38 MAPK activity were abolished in cell lines derived from the SCN or fibroblasts of *Per1*^*ldc*^*/ Per2*^*ldc*^ mice harboring a defective clock [33].

We discovered that *Per1* and *Per2* are required for normal accumulation of phosphorylated p38 MAPK levels in SCN and fibroblast cells, suggesting that the clock activates of p38 MAPK at specific times of the day in these cells. For cultured SCN cells in our studies, this clock control of p38 MAPK phosphorylation is consistent with that previously observed in the SCN in vivo [9]. In our SCN cultures, phosphorylated p38 MAPK levels and PER2::LUC bioluminescence peak at 40 h (Fig. 1) and 36 h (Fig. 4), respectively, after synchronization by serum shock. Based on evidence indicating that PER2::LUC expression peaks during the mid to late subjective day (CT6–12) in the mouse SCN [4], we can infer that the peak of phosphorylated p38 MAPK levels in mouse *Per2*^*Luc*^ SCN cells occurs during the late subjective day, similar to the peak of the p38 MAPK phosphorylation rhythm in hamster SCN tissue. Several studies have shown that the clock regulates the transcription of MAPK pathway components and upstream regulators as a mechanism to generate rhythms in MAPK activation. For example, evening-specific transcription of SCN circadian oscillator protein (SCOP) is thought to drive daytime rhythms in ERK MAPK activation in the SCN [51]. In Neurospora, the *os-4* gene encoding the MAPKKK of the p38 MAPK cascade, is a direct target of the positive component (WCC) of the circadian oscillator [15]. Rhythmic binding of the WCC to the promoter of *os-4* drives oscillations in *os-4* mRNA, which is necessary for the circadian rhythm of phosphorylated p38 MAPK accumulation [15]. This mechanism may be conserved in mammalian cells: RNA-seq experiments in mouse liver demonstrated that Mkk3/Mkk6, the MAPKK’s upstream of p38 MAPK, are rhythmically expressed [3, 52]. Mkk6 is a direct target of CLOCK/BMAL1 in the SCN and peripheral tissues, and Mkk3 is a target of CLOCK/BMAL1 in the kidney [3]. Thus, similar to Neurospora, circadian transcription of MAPK pathway components may play an important role in generating self-sustaining p38 MAPK activity rhythms.

In addition to being regulated by the clock, previous studies have supported that p38 MAPK alters the properties of the molecular clock. For example, chronic inhibition of p38 MAPK with SB203580 in culture chick pineal cells lengthened the period of the clock, and a pulse of inhibitor during the day led to a phase delay of the rhythm in melatonin [17]. Similarly, in *rat-1* fibroblast and C6 glioma cells, inhibition of p38 MAPK with SB203580 lengthened the period of the PER2::LUC reporter [41], and inhibition of U2OS cells with SB202190 lengthened the period of a *Bmal1-dLuc* reporter [53]. The p38 MAPK inhibitors used in these studies, SB203580 or SB202190, are known to have significant off-target effects on kinases that modulate the circadian clock, most notably CKIε [54, 55]. However, CKIε mutations that decrease its activity in hamsters shorten the period [42], suggesting that the period lengthening effects of SB203580 and SB202190 may be specific to inhibition of p38 MAPK. Consistent with this idea, we found that application of VX-745 to fibroblast cells reduced the levels of phosphorylated p38 MAPK as expected and lengthened the period of the *Bmal1-dLuc* clock reporter rhythm. In contrast, VX-745 application to SCN cells shortened the period of the PER2::LUC reporter. These data suggest that p38 MAPK has cell type-specific effects on the molecular clock, and this response is most likely through differences in downstream effectors of p38 MAPK. Cell type-specific effects on the clock were also observed with inhibition of JNK using SP600125. JNK inhibition lengthened the period of a PER2::LUC reporter in mouse SCN, pineal gland and lung explants, but abolished rhythmicity in the kidney [56]. JNK inhibition also lengthened the period of a *Bmal1-dLuc* or PER2::LUC reporter in *rat-1* fibroblasts [41, 57], and increased JNK activity shortened the period in *rat-1* fibroblasts [56]. One downstream target of p38 MAPK is the transcription factor ATF-2, which forms a heterodimer with members of the AP-1 family of proteins, including c-Jun [58] and binds to cAMP response elements or to AP-1 sequences to activate transcription. These data raise the possibility that p38 MAPK and JNK function through JUN, or other downstream AP-1 targets, to regulate tissue-specific activity of the molecular clock.

The role of p38 MAPK in inflammation [59] and as a tumor suppressor [23, 24] has suggested that inhibition of p38 MAPK might be an attractive candidate treating inflammatory disease and cancer. However, most p38 MAPK inhibitors have dose-limiting adverse effects in patients [31]. We speculated that clock-control of p38 MAPK activity may lead to time-of-day-specific differences in the efficacy of selective inhibitors, and our data support this idea. First, we demonstrated that p38 MAPK activity cycles in HA glial cells, but is arrhythmic in glioma cells with a differential elevation of levels in highly invasive IM3 glioma relative to parental C6 cells. The difference in levels of phosphorylated p38 MAPK in C6 versus IM3 glioma cells needs to be explored further, but may reflect differences in inputs to p38 MAPK pathway activation in cancer cell types with different tumorigenic or invasive properties. Despite the constant high levels of phosphorylated p38 MAPK in IM3 cells, a significant time-of-day reduction of cell invasiveness was observed with application of VX-745 corresponding to the time of trough levels of phosphorylated p38 MAPK in HA cells. The constant high level of active p38 MAPK in IM3 cells over the day likely reflects loss of circadian rhythmicity in these cells. Loss of p38 MAPK activity rhythms in IM3 cells could be due to disruption of the circadian oscillator, or due to loss of coupling between the oscillator and the p38 MAPK pathway. Consistent with a functional molecular oscillator, *Per2* and *Bmal1* mRNA levels accumulate rhythmically in IM3 cells, similar to control HA cells (Fig. 5b). Consequently, our data indicate that there is loss of coupling between the oscillator and the p38 MAPK pathway in IM3 cells. Time-of-day-specific treatment with VX-745 may restore this coupling and support rhythms in p38 MAPK activity and clock-control of invasiveness. Loss of circadian coupling may a common feature of cancer cell lines [60–62], and inducing rhythmicity with synchronizing agents such as, dexamethasone, forskolin, or heat shock, may restore coupling between the molecular clock and its outputs, and slow tumor growth [60]. Of significant interest, dexamethasone is commonly used in patients to reduce swelling in the brain surrounding glioblastoma [63], and similar to VX-745, may improve outcomes if administered at specific times of the day.

## Conclusions

Increased expression and activity of p38 MAPK has been associated with poor prognosis in certain types of cancers [27]. This has led to the development of p38 inhibitors for potential use in chemotherapy [31]. However, most, if not all, p38 MAPK inhibitors have dose-limiting adverse effects in patients. We demonstrate that p38 MAPK activity is rhythmic in normal glial cells. In contrast, p38 MAPK activity is high at all times of the day in IM3 glioma cells. We show a significant time-of-day reduction of cell invasiveness in IM3 cells with application of the specific p38 inhibitor VX-745 [43] corresponding to the time of trough levels of phosphorylated p38 MAPK in normal glial cells. We conclude that the timing of administration of p38 MAPK inhibitors provides a promising avenue to pursue in the treatment of cancer to improve patient outcomes.

## Additional files

Additional file 1: Full western blots of gels from Fig. 1. (DOXC 243 kb)

Additional file 2: Full western blots of gels from Fig. 5. (DOXC 200 kb)

## Abbreviations

bHLH: Basic helix-loop-helix
ERK: Extracellular signal-regulated kinase
HA: Human astroglia
JNK: C-Jun N-terminal kinase
MAPK: Mitogen activated protein kinase
Phospho-p38 MAPK: Phosphorylated p38 MAPK
SCN: Suprachiasmatic nucleus
SEM: Standard error of the mean
