# Correction to: inhibition of p38 MAPK activity leads to cell type-specific effects on the molecular circadian clock and time-dependent reduction of glioma cell invasiveness

**DOI:** 10.1186/s12885-018-5238-0

**Published:** 2019-01-23

**Authors:** Charles S. Goldsmith, Sam Moon Kim, Nirmala Karunarathna, Nichole Neuendorff, L. Gerard Toussaint, David J. Earnest, Deborah Bell-Pedersen

**Affiliations:** 1 Interdisciplinary Program in Genetics, Texas A&M University, College Station TX, Texas 77843 USA; 2 Department of Biology, Texas A&M University, College Station, Texas, TX 77843 USA; 3 Department of Neuroscience and Experimental Therapeutics, Texas A&M, Health Science Center, College of Medicine Bryan, Texas, TX 77807-3260 USA; 4 Center for Biological Clocks Research, Texas A&M University, College Station, Texas, TX 77843 USA; 5 Interdisciplinary Program in Neuroscience, Texas A&M University, College Station, Texas, TX 77843 USA

**Correction to: BMC Cancer (2018) 18:43**

**http://doi.org/10.1186/s12885-017-3896-y**

Following publication of the original article [1], we have been notified that the tagging of one of the author names was done incorrectly in the XML version of the paper. The online and pdf versions of this paper are not affected by the change. Original and corrected tagging can be seen below. The original article has been corrected.

- Incorrect name tagging:
  Last Name: Gerard Toussaint.
  First Name: L.
- Correct author name tagging:
  Last Name: Toussaint.
  Middle Name: Gerard.
  First Name: L.
