# Supplementary material for: Inhibition of p38 MAPK activity leads to cell type-specific effects on the molecular circadian clock and time-dependent reduction of glioma cell invasiveness
Source: BMC Cancer. 2018 Jan 10;18:43. doi: 10.1186/s12885-017-3896-y (PMC5761097; doi:10.1186/s12885-017-3896-y)

**Additional File 1**: Full western blots of gels from Figure 1. WT and *Per1_ldc_/Per2_ldc_* SCN and fibroblast cells were probed with phospho-p38 (pp38) and total p38 antibodies for the indicated times (h) after serum shock. Molecular weight (kDa) markers (MW) are shown: WT SCN (Invitrogen SeeBlue Plus2 marker), WT fibroblast and *Per1_ldc_/Per2_ldc_* SCN and fibroblast (Precision Plus Protein™ All Blue Prestained Protein Standards). The molecular weight of p38 MAPK is 41.3 kDa. Arrows denote pp38 and p38.


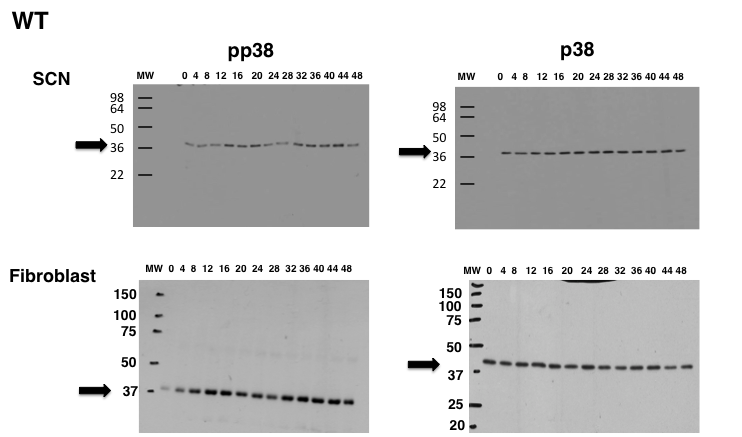


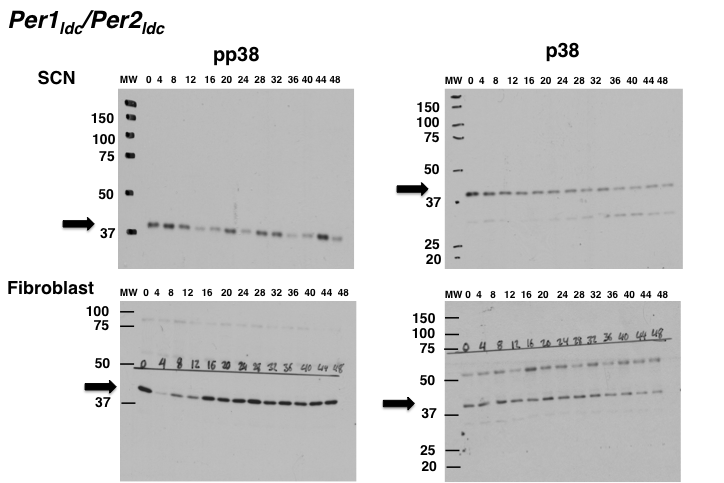

Supplement: Supplementary file 1 — Full western blots of gels from Fig. 1. (DOXC 243 kb) [file 12885_2017_3896_MOESM1_ESM.docx]
