# Supplementary material for: Inhibition of p38 MAPK activity leads to cell type-specific effects on the molecular circadian clock and time-dependent reduction of glioma cell invasiveness
Source: BMC Cancer. 2018 Jan 10;18:43. doi: 10.1186/s12885-017-3896-y (PMC5761097; doi:10.1186/s12885-017-3896-y)

**Additional File 2**: Full western blots of gels from Figure 5. HA, C6, and IM3 cells were probed with phospho-p38 (pp38) and total p38 antibodies for the indicated times (h) after serum shock. Molecular weight (kDa) markers (MW) are shown (Thermo Scientific Prestained Protein Molecular Weight Marker). The molecular weight of p38 MAPK is 41.3 kDa. Arrows denote pp38 and p38.


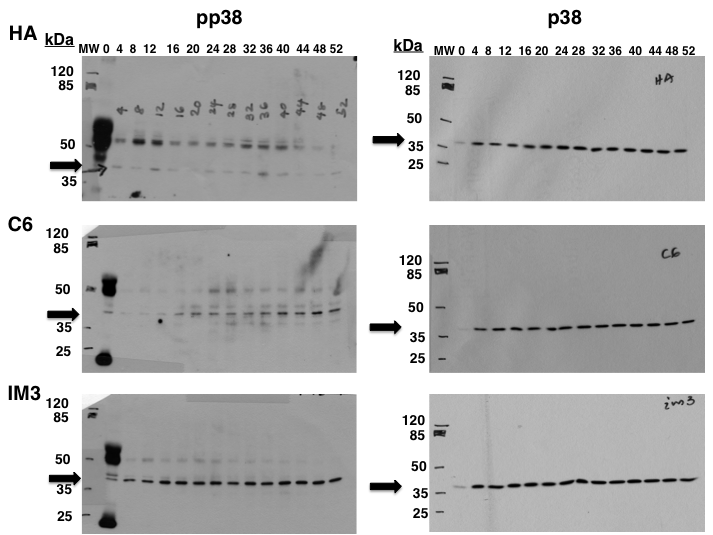

Supplement: Supplementary file 2 — Full western blots of gels from Fig. 5. (DOXC 200 kb) [file 12885_2017_3896_MOESM2_ESM.docx]
